# Annotations

**Published:** 1911-02-25

**Authors:** 


### Annotations.

### A Notice.

With reference to a paragraph in the issue of The Hospital for December 17, 1910, entitled "An Advance Announcement," in which comments were made upon an advertisement of a new book by Mr. H. C. Ross, we desire to state that we are fully satisfied that Mr. Ross was in no way responsible for, or had knowledge of, the advertisement in question, and to withdraw unreservedly anything in the paragraph which might be calculated to give a contrary impression. We wish, at the same time, to express our great regret to Mr. Ross for any injury or annoyance that our comments may have caused him.

### The Future of the White Races.

A statistical expert has recently published in Germany some significant figures regarding the population and religions of the world. He gives the total population of the world as 1,544,500,000, of whom 534,940,000, or a little over one-third, are taken to be Christians. The Christian races mainly inhabit Europe, North and South America, and Australasia, while the non-Christian races practically monopolise Asia and Africa. What is disquieting is that the birth rates in the countries inhabited by Christians show a uniform and rapid decline, while there is nothing to show a corresponding decline in Asia and Africa. Thus, if the German figures be correct, we have 1,000,000,000 coloured non-Christians living on the earth side by side with 534,000,000 white Christians. Even supposing that the birth rate were equal in both classes, the coloured non-Christians, other conditions being equal, would soon exercise an overwhelming numerical preponderance, for population increases in geometrical progression. But when we find that the white class, which is only half the size of the coloured class, is further handicapped by a steadily declining birth rate, the future of the white races does not seem by any means rosy.

### The Meat and Milk Supply.

A new monthly review has just come into being entitled *The Journal of Meat and Milk Hygiene*, devoted to securing for Great Britain and Ireland an efficient supply of meat and milk. In an apologetic foreword, the editor hopes that the new journal, dealing as it does with one particular branch of public health work, will occupy a position of its own, without thereby detracting from the value of its contemporaries. We are relieved to find that the promoters of the publication have no intention of starting an association or holding an annual congress. They acknowledge their indebtedness to their French contemporary, *L'Hygiène de la Viande et du Lait*, in coming to a decision as to the form the new journal should take. The first number contains three original articles, respectively on "Meat Poisoning," "The Occurrence of Actinomycosis in Cows' Udders," and on "The Public Slaughter-house System of Scotland," in addition to notes and abstracts, annotations, public health reports, legal and official matters, reviews of books and current literature, etc. It forms an interesting publication, and can be had from the publishers, Messrs. John Bale, Sons, and Danielsson, Limited, for a subscription of 12s. per annum, post free.

### "Truth's" Cautionary List.

Not only Pantagruel delighted in "discovering truths as he lay in bed," for the encyclopædic student of fiction will possibly be able to parallel his case with several others drawn from classical authors. It is a truism that what happened to a fictitious character years ago is just as likely to happen to people nowadays. The moral of all this is that we are not too far advanced to discover truths when we are lying abed, and for that reason it is worth while putting in a claim for *Truth's* cautionary list as an example of useful and entertaining reading to the convalescent patient! This year's edition, which may, like its predecessors, be obtained from any bookstall for the very modest and thoroughly well spent shilling, is a mine of instruction. No doubt the philosopher will find it all very saddening, but we confess to a twinge of enjoyment when we perused it. "We're there to be made game of," remarks the stern gentleman in one of Grillparzer's plays, and this fact the bucket-shop keeper, the home-employment trickster, the quack, the tallyman, and the dozen odd varieties of shady swindlers who are pilloried in this annual are fully aware of. It is therefore a trifling compensatory pleasure to be able to look at their tricks and swindling, which on occasion are vastly amusing—to those who have not suffered from them—with a smile of superiority. When we take up *Truth's* Cautionary List we perfervidly thank providence that we are not as other men (and women) are. Our professional training has made us keen to see and expose the quackery in mixtures we do not prescribe and systems of treatment we do not ourselves employ; but are we all so intelligent that we are never "bitten" by the other kinds of sharks that infest our civilisation? A perusal of the list suggests some unpleasant thoughts, which are a salutary set-off against the entertainment we may derive from reading it. At the same time it is worth while that we should for some moments at least occupy ourselves with such things, and for that reason we heartily commend the list to every practitioner. We repeat what we have said on more than one occasion: it would be an excellent thing if every medical man invested in a copy of this book and placed it on the table in his waiting-room. We add this year another suggestion: it will be an equally good thing if some really charitable person invested in a few hundred copies and sent them to the secretaries of the various voluntary hospitals throughout the country with the request that they should be placed in the ward library. There are many convalescent patients who will take up such a book and pass, like Pantagruel, an instructive hour in learning truth.

